# Calaxin is required for asymmetric bend initiation and propagation in sperm flagella

**DOI:** 10.3389/fcell.2023.1136404

**Published:** 2023-03-16

**Authors:** Kogiku Shiba, Shoji A. Baba, Eiji Fujiwara, Kazuo Inaba

**Affiliations:** ^1^ Shimoda Marine Research Center, University of Tsukuba, Shimoda, Japan; ^2^ Ochanomizu University, Otsuka, Japan; ^3^ Documentary Channel Co. Ltd., Tsurugashima, Japan

**Keywords:** dynein, caged ATP, calcium ion, cilia, sperm motility

## Abstract

Regulation of waveform asymmetry in flagella is critical for changes in direction when sperm are swimming, as seen during the chemotaxis of sperm towards eggs. Ca^2+^ is an important regulator of asymmetry in flagellar waveforms. A calcium sensor protein, calaxin, is associated with the outer arm dynein and plays a key role in the regulation of flagellar motility in a Ca^2+^-dependent manner. However, the underlying mechanism of regulating asymmetric waves by means of Ca^2+^ and calaxin remains unclear. To clarify the calaxin-dependent mechanism for generating Ca^2+^-dependent asymmetric flagellar waveforms, we analyzed the initial step of flagellar bend formation and propagation in the sperm of the ascidian *Ciona intestinalis*. Our experiment used demembranated sperm cells, which were then reactivated by UV flash photolysis of caged ATP under both high and low Ca^2+^ concentrations. Here, we show that initial bends in the flagella are formed at the base of the sperm and propagate towards the tip during waveform generation. However, the direction of the initial bend differed between asymmetric and symmetric waves. When a calaxin inhibitor (repaglinide) was applied, it resulted in the failure of asymmetric wave formation and propagation. This was because repaglinide had no effect on initial bend formation, but it significantly inhibited the generation of the subsequent bend in the reverse direction. Switching of dynein sliding activity by mechanical feedback is crucial for flagellar oscillation. Our results suggest that the Ca^2+^/calaxin mechanism plays an important role in the switching of dynein activity from microtubule sliding in the principal bend into the suppressed sliding in the reverse bend, thereby allowing the sperm to successfully change direction.

## 1 Introduction

Changes in the bending pattern of cilia and flagella are important for the regulation of cell movement and extracellular fluid flow (Inaba, 2015). In particular, the waveform of the sperm flagellum is composed of two bends: a principal bend (P-bend) and a reverse bend (R-bend) (Goldstein, 1977; Brokaw, 1979; Gibbons and Gibbons, 1980; Inaba and Shiba, 2018). When the radii of curvature of the two opposite flagellar bends are almost the same, sperm cells generate symmetric waveforms and swim straight. However, when the radius of curvature of the P-bend is much larger than that of the R-bend, sperm cells generate highly asymmetric waveforms, resulting in circular movements. The regulation of asymmetry in sperm flagellar waveforms is critical for changes in sperm swimming direction, as seen during sperm chemotaxis towards the eggs (Miller, 1985; Yoshida and Yoshida, 2011; Wachten et al., 2017).

Ca^2+^ plays a key role in the regulation of asymmetry in flagellar waveforms. For instance, the flagellar waveforms in demembranated models of sea urchin sperm cells change from symmetric to asymmetric with increasing Ca^2+^ concentrations (Brokaw, 1979; Gibbons and Gibbons, 1980). Such a change is implied in egg fertilization, as Ca^2+^ bursts induce highly asymmetric waveforms during sperm chemotaxis, thereby inducing the turning of sperm towards the egg in both ascidians and sea urchins (Bohmer et al., 2005; Wood et al., 2005; Shiba et al., 2008; Guerrero et al., 2010). However, much of the Ca^2+^-dependent mechanism that spatiotemporally regulates the formation and propagation of asymmetric waves remains unknown.

We previously identified a Ca^2+^-binding axonemal protein, calaxin, in the sperm of the ascidian *Ciona intestinalis* (recently renamed *C. intestinalis* type A or *Ciona robusta*). Calaxin is a member of the neuronal calcium sensor (NCS) family and directly interacts with the outer arm dynein on all nine doublet microtubules from the base to the tip of the flagellum (Mizuno et al., 2009). Inhibition of calaxin with the specific NCS inhibitor, repaglinide, suppresses the propagation of asymmetric flagellar waveforms required for chemotactic turn; however, a transient increase in intracellular Ca^2+^ during the turning movement normally still occurs (Mizuno et al., 2012). Although this suggests an important role of calaxin in the regulation of asymmetric flagellar bending, it is not completely understood how calaxin contributes to the formation and regulation of asymmetric waves. Calaxin homologs are well conserved among metazoans and some fungi (Inaba, 2015). Recent structural study using cryoelectron microscopy have identified the precise location of calaxin near outer dynein arms within the bovine respiratory cilia (Gui et al., 2021), suggesting the critical role of calaxin on dynein regulation and ciliary and flagellar motility.

Flagellar bending is associated with the coordination of microtubule sliding by multiple axonemal dyneins. The switching of dynein activity across the axoneme is thought to be controlled through the regulation of the central pair (CP) and radial spoke (RS) (Sale, 1986; Hayashi and Shingyoji, 2008; Lin and Nicastro, 2018). Mutual activation of opposite dyneins across the central pair in the axoneme induces flagellar oscillations (Lindemann and Lesich, 2010; Inaba, 2011; Lindemann, 2011; Shingyoji, 2013; Lin and Nicastro, 2018). The propagation of asymmetric flagellar waves is regulated in a Ca^2+^- dependent manner. In fact, Ca^2+^-binding proteins, including centrin and calmodulin, along with calmodulin-binding proteins, are localized as components of some inner arm dyneins or of the CP/RS (Smith and Yang, 2004; Wargo et al., 2005; Yamamoto et al., 2013; Zhao et al., 2019). The aim of our study is to determine how the Ca^2+^ sensor of the outer arm dynein, calaxin, contributes to the propagation of asymmetric waves.

To this end, we performed experiments using caged ATP to capture instantaneous images of the initial process for the formation and propagation of asymmetric waves and examined the effects of a calaxin inhibitor on these steps. We show that calaxin is not required for the formation of an initial P-bend at the flagellar base but is indispensable for the regulation of R-bend in the reverse direction. Our results suggest that Ca^2+^/calaxin promotes the formation, growth and propagation of asymmetric flagellar waves by attenuating dynein-driven microtubule sliding in the reverse bend *via* mechanical feedback from the adjacent P-bend.

## 2 Materials and methods

### 2.1 Materials

The ascidian *C. intestinalis* (type A; also called *C. robusta*) was collected from Onagawa Bay near the Onagawa Field Research Center, Tohoku University or obtained from the National BioResource Project for *Ciona* (http://marinebio.nbrp.jp/). Animals were kept in aquaria under constant light for accumulation of gametes without spontaneous spawning. Semen samples were collected by dissecting the sperm duct and kept on ice until use.

### 2.2 Chemical and solutions

Ca^2+^-free sea water (CFSW) contained 478.2 mM NaCl, 9.39 mM KCl, 48.27 mM MgCl_2_, 2.5 mM EGTA and 10 mM Hepes-NaOH (pH 8.0). Sperm demembranation solution contained 0.2 M potassium acetate, 1 mM MgSO_4_, 2.5 mM EGTA, 20 mM Tris-acetate (pH 7.8), 0.04% Triton X-100 and 1 mM DTT. Pre-incubation buffer contained 0.15 M potassium acetate, 2 mM MgSO_4_, 2.5 mM EGTA, 39 μM (pCa10) or 2.51 mM (pCa5) CaCl_2_, 50 mM Tris-HCl (pH8.0) and 1 mM DTT. Reactivation solution was the same as pre-incubation buffer but with 2 mM ATP. Free Ca^2+^ concentration was assessed by CALCON (http://www.bio.chuo-u.ac.jp/nano/calcon.html). Caged ATP was purchased from Dojindo (Kumamoto, Japan) dissolved in Pre-incubation buffer. Repaglinide was purchased from Sigma-Aldrich (St Louis, MO) and dissolved in dimethyl sulfoxide (DMSO). Theophylline from Sigma-Aldrich was dissolved in CFSW. Other reagents were of analytical grades.

### 2.3 Preparation of demembranated sperm

Demembranation and reactivation of *C. intestinalis* sperm were performed as described previously with some modifications (Mizuno et al., 2012). Semen was suspended in 100 volumes of CFSW containing 1 mM theophylline in CFSW to activate motility and incubated for 5 min at 25°C. Sperm were demembranated with 10 volumes of demembranation solution incubated for 3 min at 25°C. Demembranated sperm were kept for several minutes in pre-incubation buffer with or without 1 mM caged ATP until use. Demembranated sperm incubated with caged ATP were reactivated by UV irradiation. Demembranated sperm without caged ATP were reactivated with the same volume of the reactivation buffer containing 0.1–2 mM Mg-ATP (Final Mg-ATP concentrations were 0.05–1 mM). DMSO (control) or Repaglinide was added to the demembranation solution, pre-incubation buffer and reactivation buffer. DMSO concentration was kept below 0.5% in all experiments.

### 2.4 Experimental system

Movements of demembranated sperm incubated with caged ATP were observed under an inverted microscope with phase optics (IX71, Olympus, Tokyo, Japan) with a ×20 objective and recorded with a high-speed CCD camera (HAS220, Detect, Tokyo, Japan). Flagellar waveforms were captured using a red-LED (Edison 3WStar, Taipei, Taiwan) and a laboratory-made LED stroboscopic illumination system synchronized with the exposure signals from the high-speed camera. Images were taken at a frame rate of 200 fps with 0.2 msec pulse from the red LED. A UV-LED (NS365L-3SVR, 365 nm, Nitride Semiconductors, Tokushima, Japan) was used for the photolysis of caged ATP. A mercury lamp in the lamp house of the microscope was replaced by a laboratory-made LED holder with UV-LED. A laboratory-made LED controller generated a 150 msec pulse for UV irradiation and triggered a recording cue signal on the high-speed camera (HAS220) which enables to record images 0.25 s before and 2.25 s after UV irradiation. To measure the flagellar beat frequency of sperm reactivated with Mg-ATP, flagellar waveforms were observed under an upright microscope with phase optics (BX51, Olympus) with a ×20 objective and recorded with a high-speed CCD camera (HAS-D3, Detect) at 500 fps.

### 2.5 Analysis of flagellar waveforms

The flagellar beat frequency and the flagellar curvature were analyzed using Bohboh software (Bohbohsoft, Tokyo, Japan). Individual images of sperm flagella were tracked automatically, and their curvatures calculated based on the method of Baba and Mogami (1985). Pseudocolor-maps of the flagellar curvature along the distance and time were created using by gnuplot 5.0 (http://www.gnuplot.info/).

### 2.6 Statistical analysis

All experiments were repeated at least three times with three different specimens. Data is expressed as means ± SE. Statistical significance was calculated using Student’s t-test; *p* < 0.05 was considered significant.

## 3 Results

To elucidate the mechanism underlying the formation and propagation of asymmetric flagellar waves, we performed experiments using caged ATP to capture initial bend formation and subsequent propagation. Specifically, *Ciona* sperm were demembranated with Triton X-100 and incubated with 1 mM of caged ATP. Reactivation of motility was induced by UV flash photolysis of caged ATP. This method released the ATP from its caged compound within a few seconds by a UV flash (McCray et al., 1980; Goldman et al., 1982). To record the changes in sperm flagellar waveforms upon the UV flashing, we used a phase-contrast microscope with a red LED for illumination and captured the images using a high-speed camera, to which the LED pulse was synchronized. UV flashes were applied through the objective lens by a UV LED located in the lamp house.

**FIGURE 1:**
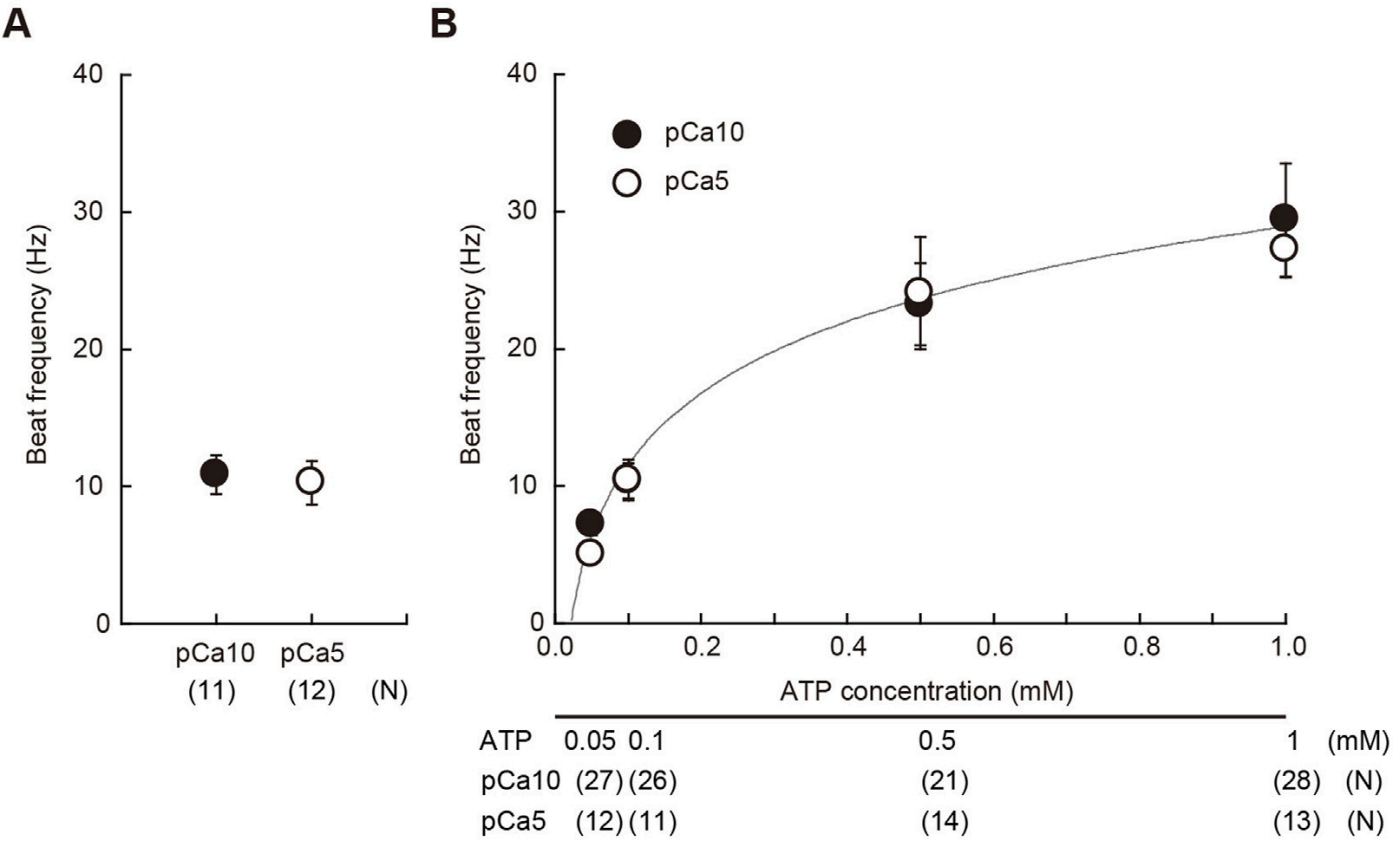
Estimation of the concentration of ATP released from caged ATP. **(A)** Beat frequency of demembranated *Ciona* sperm incubated with 1 mM caged ATP and reactivated by a 150 ms UV flash. N = 11 (pCa10), and N = 12 (pCa5). **(B)** Beat frequency of demembranated and reactivated *Ciona* sperm by various concentrations of Mg-ATP in the absence of caged ATP. Closed and open circles show Ca^2+^ concentrations in the reactivation solutions at pCa10 and pCa5, respectively. N = 13–28 from three different experiments. Values are expressed as mean ± S.D.

After the UV flashing, we estimated the concentration of ATP released from caged ATP by measuring the flagellar beat frequency. The beat frequency of demembranated sperm, which had been reactivated with 1 mM of caged ATP and a 150 ms UV flash was estimated to be approximately 10 Hz (Figure 1A). Notably, sperm beat frequency did not significantly differ between solutions with low (10^−10^ M Ca^2+^; pCa10) and high (10^−5^ M Ca^2+^; pCa5) Ca^2+^ concentrations. In this study we used two different concentrations of Ca^2+^ to observe typical asymmetric and symmetric flagellar waves during chemotactic turn. To estimate the concentration of ATP release from caged ATP, we measured the beat frequency of demembranated sperm that were reactivated by various concentrations of Mg-ATP (Figure 1B). The flagellar waveform became more asymmetric at pCa5 than at pCa10, but no significant difference was observed in the beat frequency between two Ca^2+^ conditions within the range of 0.05–1 mM Mg-ATP present. From the calibration curve, we estimated that approximately ∼0.1 mM of ATP was released under these conditions by the photolysis of caged ATP.

**FIGURE 2:**
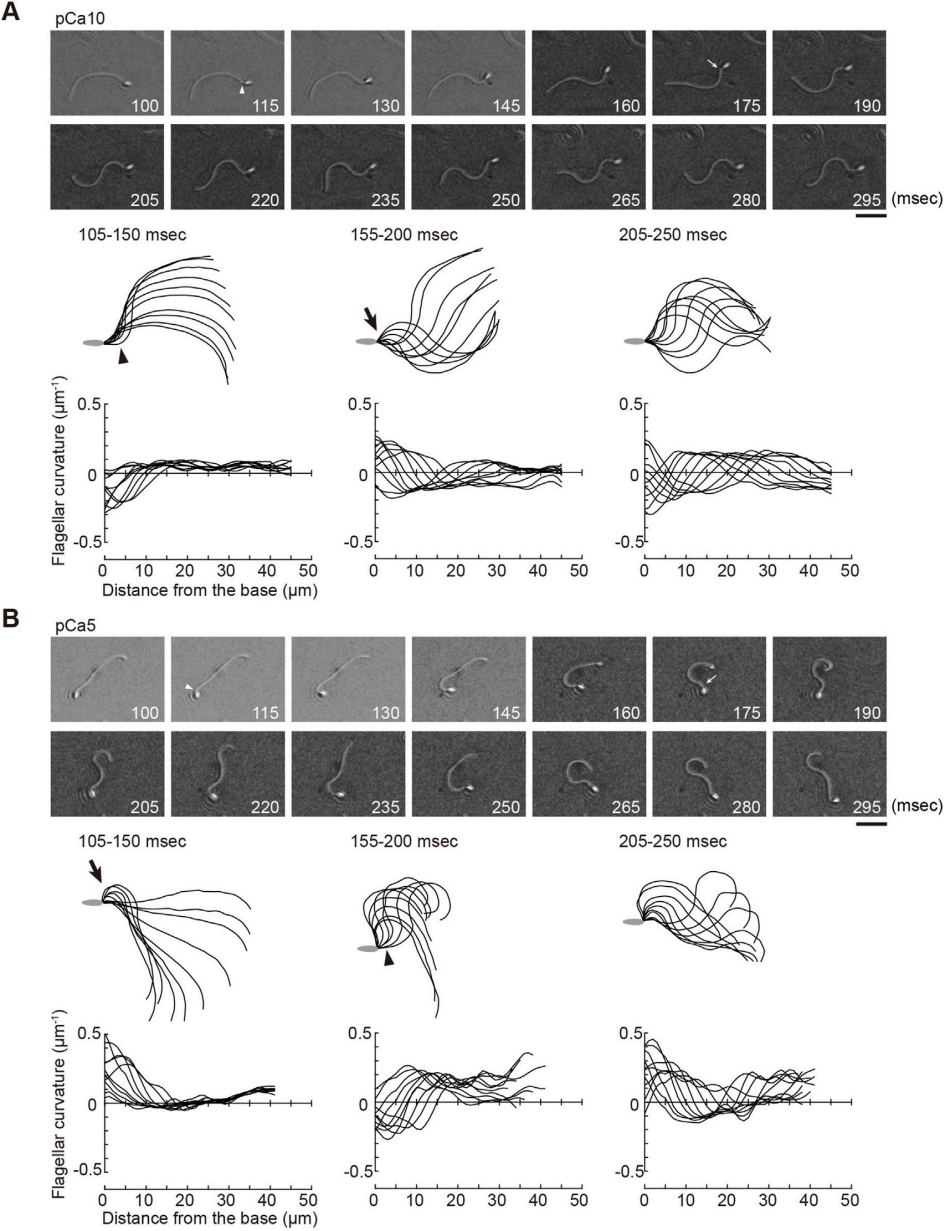
Generation of symmetric and asymmetric flagellar waveforms in *Ciona* sperm by photolytic release of caged ATP. Sperm cells were demembranated and incubated in the reactivation solution with caged ATP at low (pCa10; **(A)**) or high (pCa5; **(B)**) Ca^2+^ concentrations. Release of ATP was triggered by UV flash. Upper panel: sequential images of sperm flagellar waveforms at 15 ms-intervals from 100 ms after the UV flash. Arrow heads and arrows indicate the initial bend and the second bend, respectively. Scale bar, 20 μm. Middle panel: overwritten images of demembranated sperm at 5 ms intervals during the periods of time (105–250 ms) after release of ATP. Arrow heads and arrows indicate the initial bend and the second bend, respectively. Lower panel: changes of flagellar curvature occurring 105–250 ms after the UV flash are plotted against the distance from the base of flagellum. Ten waveforms produced at 50 ms are overwritten. Symmetric and asymmetric waveforms were generated in low calcium concentrations (pCa10, **(A)**) and high calcium concentrations (pCa10, **(B)**), respectively. One typical sperm example from the all experiments at least three times with three different specimens was shown.

**FIGURE 3:**
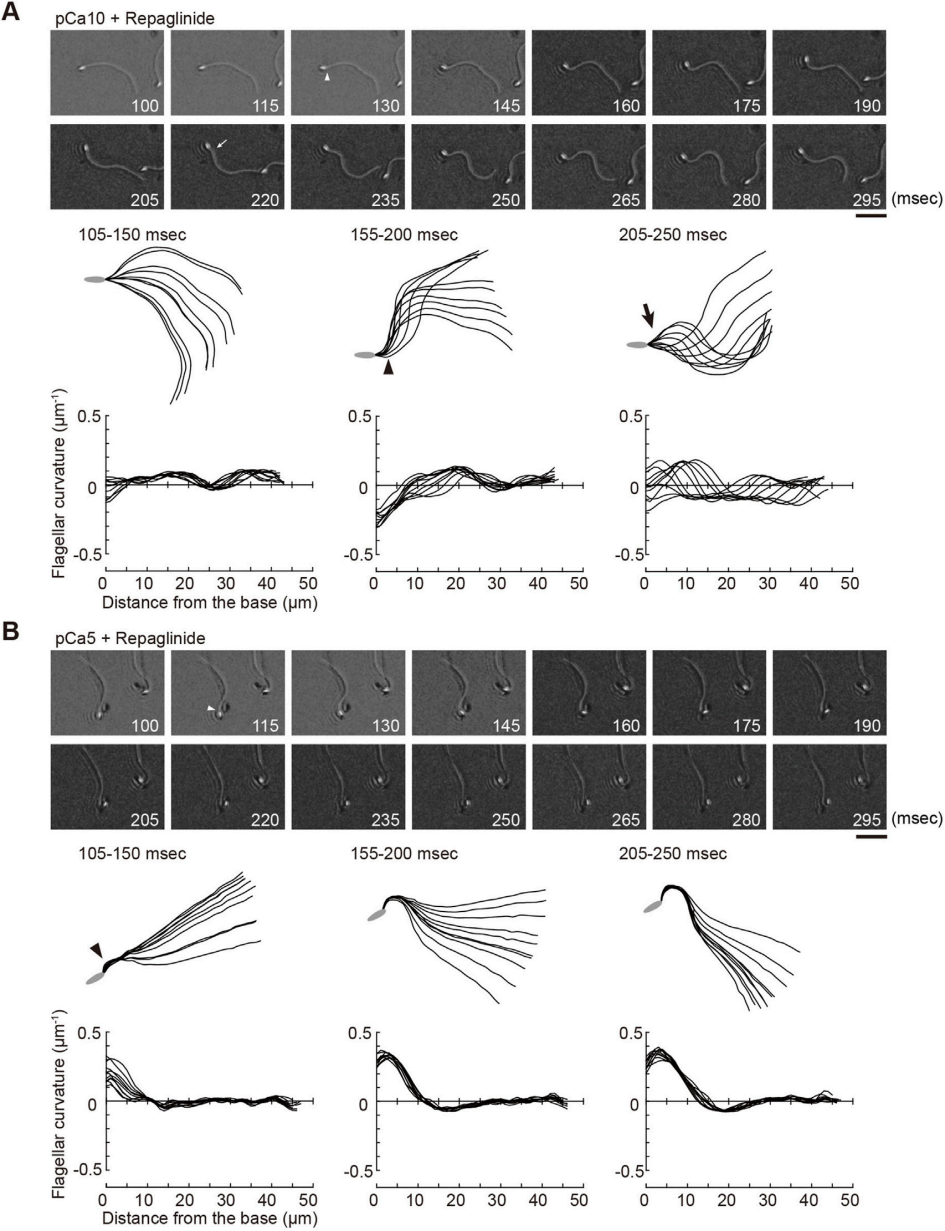
Effect of a calaxin inhibitor on the formation and propagation of asymmetry waveforms induced by photolytic release of caged ATP at high calcium concentrations. Sperm cells were demembranated and incubated in the reactivation solution with caged ATP at low (pCa10; **(A)**) or high (pCa5; **(B)**) Ca^2+^ concentrations with 150 μM repaglinide. Release of ATP was triggered by UV flash. Upper panel: sequential images of sperm flagellar waveforms at 15 ms-intervals from 100 ms after the UV flash. Arrow heads and arrows indicate the initial bend and the second bend, respectively. Scale bar, 20 μm. Middle panel: overwritten images of demembranated sperm at 5 ms intervals during the periods of time (105–250 ms) after release of ATP. Arrow heads and arrows indicate the initial bend and the second bend, respectively. Lower panel: changes of flagellar curvature occurring 105–250 ms after the UV flash are plotted against the distance from the base of flagellum. Ten waveforms produced at 50 ms are overwritten. One typical sperm example from the all experiments at least three times with three different specimens was shown.

Next, we analyzed the process for the formation and propagation of flagellar waveforms upon ATP release. Low and high Ca^2+^ concentrations induced symmetric and asymmetric waveforms, respectively (Brokaw, 1979; Gibbons and Gibbons, 1980; Inaba and Shiba, 2018). By using photolysis for the controlled induction of dynein-microtubule sliding, we captured the initial process of bend formation and propagation of symmetric and asymmetric waves. All the regions of the flagellum from the base to the tip were examined under a microscope, and it was observed that the formation and propagation of flagellar bends occurred in one plane without any helical configuration (Figure 2; Figure 3).

Ascidian spermatozoa reactivated under low Ca^2+^ concentrations form a slightly asymmetric waveform and swim in a circle with a large radius. On the other hand, sperm reactivated at higher Ca^2+^ concentrations form a highly asymmetrical waveform, resulting in a circular motion with a small radius (Brokaw, 1987). In our experimental system using caged ATP, ATP release by photolysis at pCa10 induced the formation of the initial bend at the base of the flagellum 100–150 ms after the UV flash (Figure 2A; Table 1, Supplementary Video S1). As the bend propagated towards the flagellar tip, the formation of a second bend started to grow within 200 ms. Responses with similar time courses were observed in the formation and propagation of flagellar waves at pCa5, although the curvature of the initial bend was larger than that of the second bend (Figure 2B; Table 1, Supplementary Video S1).

**TABLE 1:** Properties of flagellar bends generated by the photolysis of caged ATP.

	DMSO (control)		Repaglinide	
	pCa10	pCa5	pCa10	pCa5
Maximum curvature (μm^−1^)	0.211 ± 0.006^††^	0.320 ± 0.021^**^	0.127 ± 0.016^***^	0.279 ± 0.025^*^
Curvature of initial bend (μm^−1^)	−0.164 ± 0.011	0.279 ± 0.020	−0.111 ± 0.037	0.185 ± 0.028^†^
Time by initial bend formation (ms)	139.00 ± 4.56	138.33 ± 5.23	190.00 ± 9.67^***^	168.13 ± 12.68
Beat period (ms)	75.00 ± 4.69^†^	88.89 ± 3.75^*^	81.11 ± 3.75	16.62 ± 1.21

Values are means ± S.E. N = 5–10. Flagellar bending was analyzed from successive 50 waveforms at 5 ms intervals. The curvature was determined at 5 μm from the base. *Significant at *p* < 0.05, **p < 0.01 or ***p < 0.001 (Student’s t-test) as compared with DMSO, at pCa10. †Significant at *p* < 0.05, ††*p* < 0.01 or †††*p* < 0.001 (Student’s t-test) as compared with DMSO, at pCa5. Curvature of initial bend was compared between DMSO, and Repaglinide only.

We measured the curvatures along the flagellum, when flagellar oscillation was completely induced 200 ms after UV irradiation. We obtained the maximum and minimum flagellar curvatures in one flagellar beat cycle. In this study the bend showing the larger or smaller absolute value of curvature was defined as the P-bend or R-bend, respectively. We confirmed that the bend with larger curvature corresponded to the bend outside the sperm swimming path, which was originally defined the P-bend (Gibbons and Gibbons, 1972; Brokaw, 1979; Shingyoji, 2013). The curvature of the P-bend was plotted as a positive value and that of the R-bend was plotted as a negative value (Figures 2A, B bottom). Notably, we found that the initial bends of sperm reactivated at pCa10 consisted mostly of R-bends (Figure 2A). In contrast, the initial bends formed in the sperm reactivated at pCa5 were almost exclusively P-bends (Figure 2B). These results suggest that the initial bends are formed at the base in both symmetric and asymmetric waves but their directions are different depending on the high and low Ca^2+^ concentrations.

We previously showed that calaxin is an important factor that regulates the propagation of asymmetric waveforms (Mizuno et al., 2012). Therefore, we next examined the role of calaxin in the formation, growth, and propagation of a bend during the initial generation of asymmetric flagellar waves. We used an inhibitor of the NCS family proteins, repaglinide (Okada et al., 2003), as a calaxin inhibitor since it specifically binds to calaxin and inhibits sperm chemotaxis in *Ciona* sperm flagella (Mizuno et al., 2012). Demembranated sperm cells were incubated with caged ATP and repaglinide and irradiated with UV light to induce ATP release. The process of initial bend formation and propagation was recorded at both low and high Ca^2+^ concentrations. At pCa10, both the initial and the second bends were formed normally; however, such formation occurred a little later (∼250 ms) upon repaglinide treatment when compared to that in the control (Figure 3A; Table 1, Supplementary Video S1). Moreover, the maximum curvature after UV flashing was significantly smaller in the flagella of repaglinide-treated sperm than in those of untreated sperm (Table 1). In contrast, at pCa5 in the presence of repaglinide, an initial bend was formed but did not propagate to the flagellar tip (Figure 3B, Supplementary Video S2). The flagellum continued to vibrate at a high frequency with an overall constant waveform (Supplementary Video S2). The flagellum never formed the second bend, representing a state apparently similar to the quiescent pattern that was previously reported in sea urchin sperm (Goldstein, 1979; Gibbons and Gibbons, 1980), although it might not be comparable because the latter case is observed under the condition of pCa3 and 1 mM ATP. The failure of flagellar bend propagation at the caged ATP induced activation were observed in nearly 30% of the sperm treated by repaglinide at pCa5 (Supplementary Figures S1). We have also checked the bend formation pattern in reactivated sperm by 0.1 mM and 1 mM ATP in the presence and absence of repaglinide at pCa5. As previously reported, sperm reactivated with 1 mM ATP showed waveforms attenuated by repaglinide treatment, but most sperm oscillated and showed no abnormal wave propagation. On the other hand, about 8% of the sperm reactivated with 0.1 mM ATP showed abnormal propagation after treatment with repaglinide (Supplementary Figures S1, S2).

**FIGURE 4:**
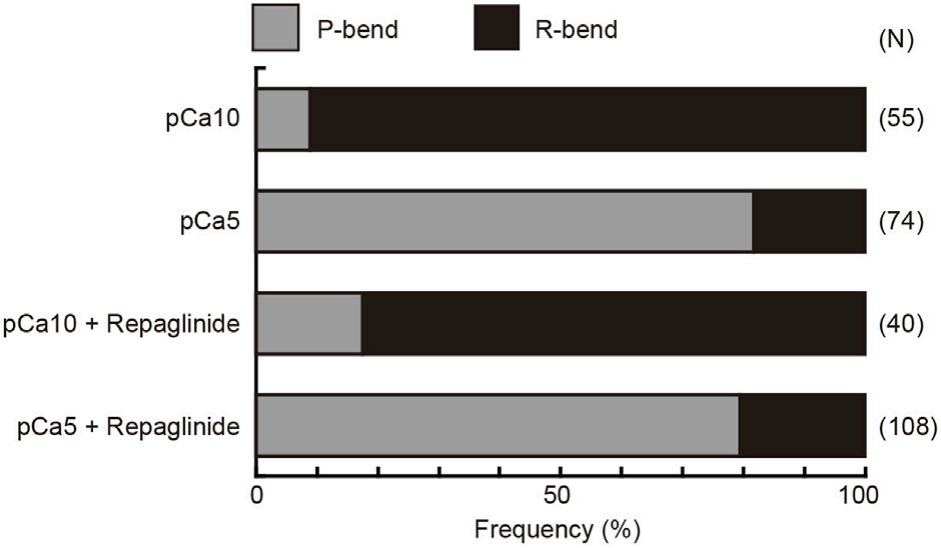
Preference for the direction of initial bend in the generation of symmetric and asymmetric flagellar waveforms. The relative frequencies of principal (P) and reverse (R) bends are shown for the initial bends formed after activation of demembranated *Ciona* sperm. Sperm was reactivated by photolysis of caged ATP in low (pCa10) or high (pCa5) Ca^2+^ concentrations in the presence of 0.5% DMSO (control) or 150 μM repaglinide. In both control and repaglinide-treated sperm, the formation of flagellar bends started with R-bends at pCa10, whereas it started with P-bends at pCa5. The number in brackets represents the number of observed spermatozoa in three different experiments.

Next, we quantified the direction of the initial bend under low and high Ca^2+^ conditions. We assigned the P- and R-bend by comparing of the curvatures of the two bends at 200 ms after UV photolysis. At low concentrations of Ca^2+^, 90.9% of the initial bend that formed upon the UV flash was observed in the reverse direction. In contrast, 81.9% of the initial bend was in the principal direction at high Ca^2+^ concentrations. The first bend direction formed in the flagella showed the same trend in the presence of repaglinide in low Ca^2+^ conditions (Figure 4). Since the flagellar wave did not oscillate in the presence of repaglinide at pCa5, it was not possible to determine the exact bend direction. However, since the curvature of the unilateral bend was as large as the P-bend formed at pCa5 in the absence of repaglinide, we assumed that the bend formed in the presence of repaglinide at pCa5 was a P-bend. These results indicate that the direction of the initial bend depends on Ca^2+^ and is unaffected by calaxin inhibition.

**FIGURE 5:**
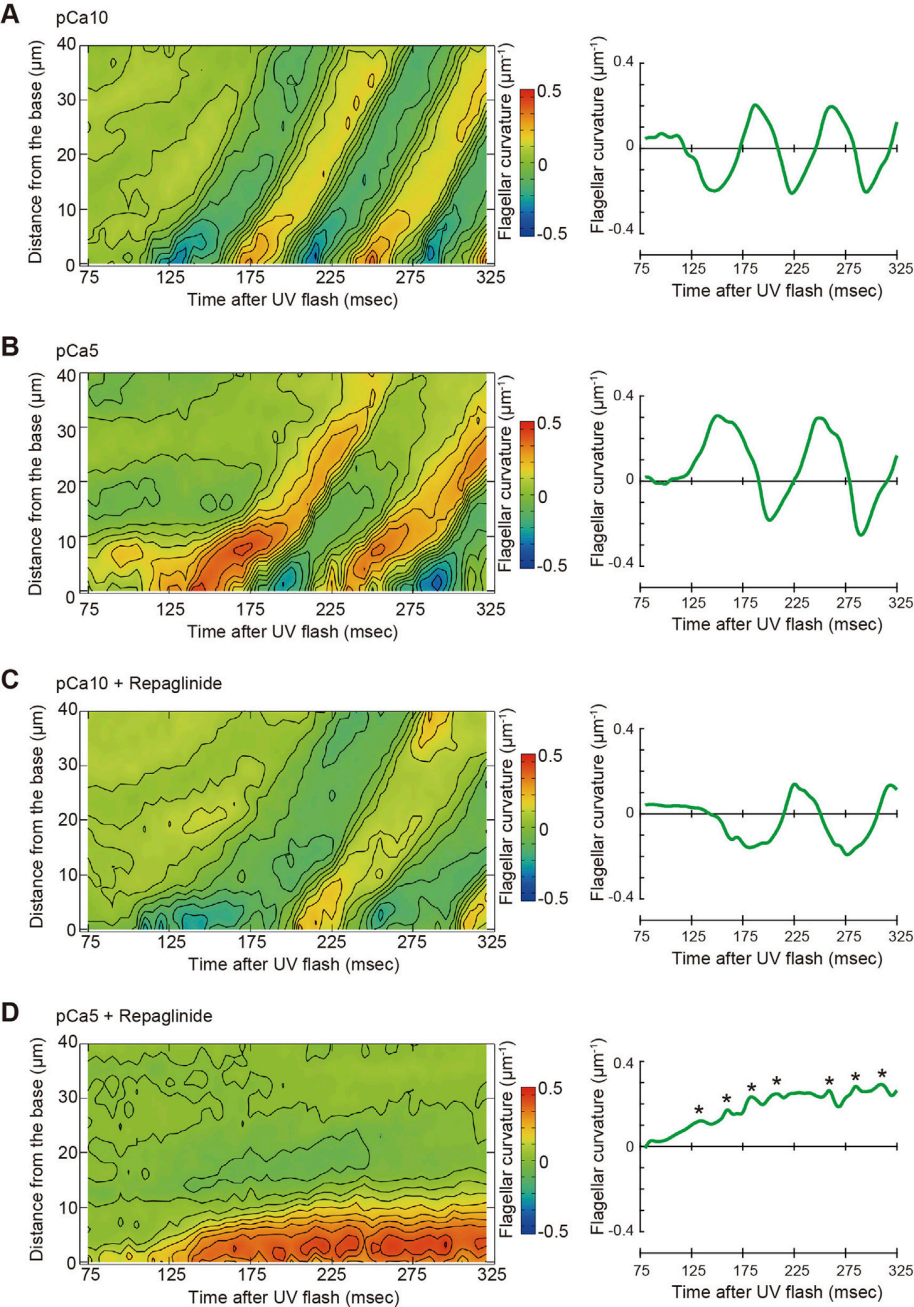
Pseudocolor maps showing spatiotemporal changes of the flagellar curvature. Left panels: the flagellar curvature of demembranated *Ciona* sperm was plotted against the distance from the base and time after the UV flash by pseudocolor mapping. Right panels: the flagellar curvature at 5 μm from the base was plotted against time after the UV flash. Sperm was reactivated through photolysis of caged ATP in low pCa10; **(A, C)** or high pCa5; **(B, D)** Ca^2+^ concentrations in the presence of 0.5% DMSO control; **(A, B)** or 150 μM repaglinide **(C, D)**.

To better understand of the process of flagellar bend formation, the changes in flagellar curvature were plotted against time and distance from the flagellar base in a three-dimensional (3D) graph (Figure 5). At pCa10, R-bends were generated at the flagellar base and propagated to the tip, followed by the generation and propagation of P-bends (Figure 5A). In contrast, P-bend formation preceded R-bend generation at pCa5 (Figure 5B). Repaglinide had little effect on the overall pattern of bend formation at pCa10, although the propagation of the R-bend proceeded more slowly and, consequently, the formation of the second bend (P-bend) was delayed (Figure 5C; Table 1). Conversely, at pCa5, repaglinide did not inhibit the generation of P-bends at the flagellar base but suppressed both the propagation and the subsequent formation of the R-bends. Under these conditions, P-bends were completely formed about ∼150 ms after UV flashing and maintained a constant curvature for up to 325 ms (Figure 5D). Although the P-bend propagation was inhibited, the high-frequency vibration of the flagellum was observed (Supplementary Video S2; Figure 5D, right). The curvature of the P-bend oscillated with a periodicity of 5–40 ms and a frequency of 75.14 ± 7.49 Hz (N = 6) (Asterisks in Figure 5D and Table 1).

## 4 Discussion

**FIGURE 6:**
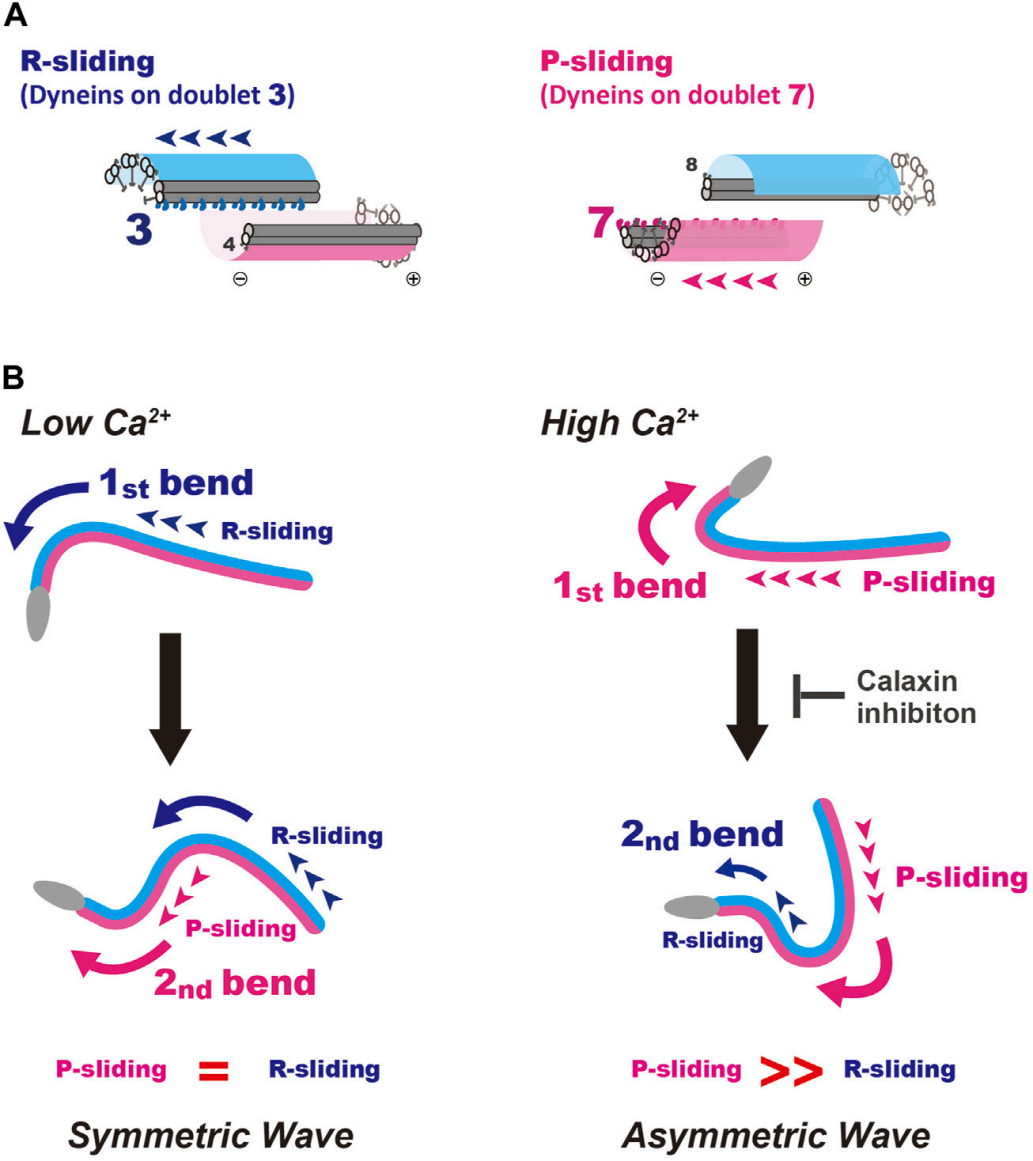
A model for the formation and propagation of the first bend and second bend in symmetric and asymmetric sperm flagellar waves. **(A)** Schematic representation for the R- and P-sliding in relation to the number of active dynein on doublet microtubule. Based on the studies of sea urchin and mammalian sperm. The formation of P-bends and R-bends is induced by the activation of dyneins on doublet 7 and 3, respectively. **(B)** A putative mechanism for initial bend formation and its propagation under low and high Ca^2+^ conditions are shown. Left, formation of a symmetric wave under low Ca^2+^ conditions. The first bend is formed by R-sliding. In turn, this R-bend induces switching of active dynein to that on the opposite side across the axoneme, resulting in P-sliding to form a P-bend. Right, generation of an asymmetric wave under high Ca^2+^ conditions. P-bend is first formed by P-sliding. In turn, this P-bend induces the switching of active dynein to that on the opposite side across the axoneme and generates suppressed R-sliding to form a R-bend with smaller curvature, resulting in the propagation of an asymmetric wave. A calaxin inhibitor, repaglinide, suppresses the generation of R-bend, possibly by inhibiting the mechanical transmission from P- to R-sliding.

Coordinated activation or inactivation of dyneins within the nine sets of doublet microtubules along the flagella control the proper formation and oscillation of flagellar bends (Inaba, 2011; King, 2018; Lin and Nicastro, 2018). The switching of dynein activity across the axoneme is particularly important for the oscillation of the sperm flagellar waveform. In sea urchin, starfish and mammalian sperm, the relationship between the directions of two opposite bends and the number of doublet microtubules with activated dyneins is understood (Sale, 1986; Mohri et al., 1987; Kanous et al., 1993; Lesich et al., 2014; Lin and Nicastro, 2018; Shingyoji, 2018) (Figure 6A). The formation of P-bends and R-bends is induced *via* the active sliding of microtubules by dyneins on doublet 7 and 3, termed P- and R-sliding, respectively (Figure 6A). We could not specify the doublet number of active dyneins in *Ciona* sperm, because of the lack of any markers observed in the axonemes of sea urchin and mammalian sperm. However, considering the structural conservation of the central apparatus throughout eukaryotic evolution (Carbajal-Gonzalez et al., 2013; Zhao et al., 2019; Leung et al., 2021) and the common effects of calaxin depletion in *Ciona* and mouse sperm (Mizuno et al., 2012; Sasaki et al., 2019), it is plausible that dyneins responsible for the P- and R-sliding would also be those on doublet 7 and 3 in *Ciona* sperm. In this study we developed a microscopic illumination system using caged ATP to capture the moment of the first bend formation. This experimental system allowed us to observe the formation and propagation of flagellar waveforms without physical disturbances such as fluid flow and mechanical stimuli (Vernon and Woolley, 1994; Tani and Kamimura, 1998; Hayashi and Shingyoji, 2008). Using this system, we found that in *Ciona* sperm, the first bend of the waves at low and high Ca^2+^ concentrations, formed at the proximal region of the flagellum, was predominantly an R-bend and P-bend respectively (Figures 2, 3). We assessed P and R bends according to the original definition (Gibbons and Gibbons, 1972; Brokaw, 1979; Shingyoji, 2013); the P-bend is the one with the larger curvature and the R-bend with a smaller curvature. Based on this definition, the first bend of symmetric waves that was formed at the proximal region of the flagellum at low Ca^2+^ was assigned as an R-bend (Figure 4). The second bend subsequently formed at the base with a larger curvature and was thus assigned as a P-bend. Following the propagation of the R-bend towards the flagellar tip, a P-bend was formed at the base of the flagellum under low Ca^2+^ conditions, presumably by switching the active dyneins to those located on the opposite side of the axoneme (Figure 6B, left). In contrast, high Ca^2+^ concentrations specifically suppresses R-sliding, resulting in waveform asymmetry (Nakano et al., 2003). Using caged ATP, we showed that *Ciona* sperm first formed a P-bend at the proximal region of the flagellum under high Ca^2+^ conditions, followed by its propagation toward the tip. Subsequently, the attenuated R-bend formed at the flagellar base (Figures 2, 3), suggesting that high Ca^2+^ suppresses R-sliding in *Ciona* spermatozoa (Figure 6B, right).

There is no clear reason for R-sliding priority in the bend formation at Low Ca^2+^. In sea urchin sperm, a sudden increase in ATP induces bend formation and propagation (Goldstein, 1979; Tani and Kamimura, 1998). Demembranated sperm models with straight flagella initially generate reverse bends (Tani and Kamimura, 1998), which coincides with our data. However, whether the initial bend starts from the P- or R-bend appears to depend on the mechanical state of the axonemes before ATP application (Goldstein, 1979). The flagella of demembranated sperm are not straight but show one large bend. This bend propagates from the formation of the initial bend (R-bend) until the distal region of the flagellum. However, bend initiation at the base is not always from the R-bend at low Ca^2+^ levels; 10%–20% flagella showed P-bend initiation at the base under the same Ca^2+^ conditions (Figure 4), indicating that the first bend formed at the base of the flagellum is equivocal.

We previously demonstrated by an *in vitro* motility assay using purified microtubules and outer arm dynein that dynein-mediated microtubule sliding was suppressed by calaxin under high Ca^2+^ conditions (Mizuno et al., 2012). This property of calaxin is essential for the propagation of asymmetric waveforms (Mizuno et al., 2012). Our present study, however, indicated the formation of the first P-bend was not affected by repaglinide under high Ca^2+^ conditions (Figure 3B), suggesting that calaxin activity is not involved in the P-sliding (Figure 6B, right). Instead, the flagellum never formed R-bend and maintained quiescence waveform (Figure 3B and Figure 5D). Therefore, calaxin is thought to be essential for the formation and propagation of R-sliding, but not for those of P-bend (Figure 6B, right). This limited participation of calaxin in the R-sliding is consistent with the observation that the R-bend propagates more slowly in the presence of repaglinide under low Ca^2+^ conditions (Figure 5C).

The mechanisms of the calaxin function in two aspects of asymmetric flagellar waves are yet unknown. First is the mechanism why the P-sliding normally occurs under high Ca^2+^ conditions, regardless of the presence or absence of repaglinide (Figure 3). Since calaxin is associated with outer arm dyneins on all nine doublet microtubules (Mizuno et al., 2009), there must be a mechanism mediating the acceleration of P-sliding by doublet 7 dynein or suppression of R-sliding by doublet 3 dynein in Ca^2+^-dependent but calaxin-independent manner. It is possible that the activity of inner arm dyneins regulated by CP/RS might affect the activity of outer arm dyneins through the regulatory protein complex connected to both the inner and outer dynein arms (Heuser et al., 2009; Pigino et al., 2011; Yamamoto et al., 2013; Oda et al., 2014). Second is the mechanism for the inhibition of R-sliding by repaglinide under high Ca^2+^ conditions. It was expected that the release of calaxin-dependent inhibition of dyneins would facilitate microtubule sliding as shown in vitro motility assay (Mizuno et al., 2012). However, repaglinide completely inhibited the formation of the R-bend and P-bend propagation, resulting in the quiescence arrest of a flagellum. Therefore, it is likely that calaxin does not simply suppress the sliding but is rather involved in the sliding at reduced velocity. This is supported by previous study (Mizuno et al., 2012) showing that R-bend formation at high Ca^2+^ (pCa5) is not inhibited in repaglinide treated sperm activated by 1 mM ATP, but propagates with attenuation. Capturing the first bend formation induced by low (0.1 mM) ATP in this study might emphasize the role of calaxin in the bend switching in flagella. The inhibition of R-sliding by repaglinide suggests that the bend formed by P-sliding at the proximal region cannot induce switching from P-sliding by doublet 7 dynein to R-sliding by doublet 3 dynein. Calaxin might be regulated by mechanical load and act a role in the generation of R-sliding in asymmetric wave by the release of calaxin-dependent inhibition of dyneins. In fact, the application of mechanical stimulations is shown to induce bend propagation in sea urchin sperm (Eshel et al., 1991; Izawa and Shingyoji, 2020). Further studies on the calaxin-mediated regulation of outer arm dyneins will shed light on this “low-gear” state sliding during the propagation of an asymmetric flagellar wave.

## Data Availability

The original contributions presented in the study are included in the article/Supplementary Material, further inquiries can be directed to the corresponding author.
