# Supplementary material for: Calaxin is required for asymmetric bend initiation and propagation in sperm flagella
Source: Front Cell Dev Biol. 2023 Mar 16;11:1136404. doi: 10.3389/fcell.2023.1136404 (PMC10061002; doi:10.3389/fcell.2023.1136404)
Supplement: Supplementary file 3 [file DataSheet1.DOCX]

Supplementary Material

Calaxin is required for asymmetric bend initiation and propagation in sperm flagella

Kogiku Shiba*, Shoji A Baba, Eiji Fujiwara and Kazuo Inaba

*** Correspondence:** Kogiku Shiba: kogiku@shimoda.tsukuba.ac.jp

# Supplementary Figures


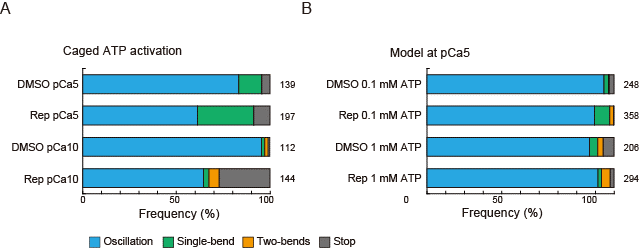


**Supplementary Figure 1.** Preference for the bend formation patterns.

**A:** The relative frequencies of the flagellar bend patterns are shown for the initial bends formed after activation of demembranated *Ciona* sperm incubated with 1 mM caged ATP and reactivated by a 150 ms UV flash. Sperm was reactivated by photolysis of caged ATP in low (pCa10) or high (pCa5) Ca^2+^ concentrations in the presence of 0.5% DMSO (control) or 150 μM repaglinide. **B:** The relative frequencies of the flagellar bend patterns are shown for the bends of demembranated *Ciona* sperm after activation by 0.1 mM or 1 mM Mg-ATP in the absence of caged ATP. Sperm was reactivated by Mg-ATP in high (pCa5) Ca^2+^ concentrations in the presence of 0.5% DMSO (control) or 150 μM repaglinide. Total sperm number is shown right on the bar.


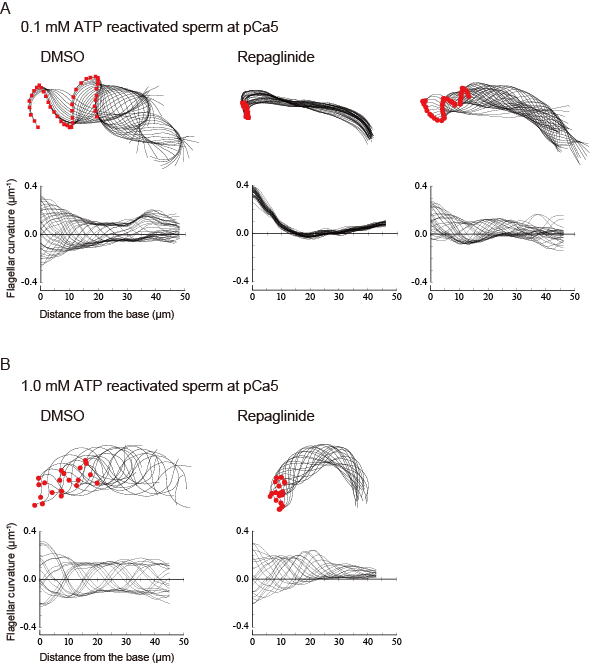


**Supplementary Figure 2.** The flagellar waveforms and curvature in demembranated *Ciona* sperm after activation by 0.1 mM (A) or 1 mM Mg-ATP (B) in the absence of caged ATP. Sperm was reactivated by Mg-ATP in high (pCa5) Ca^2+^ concentrations in the presence of 0.5% DMSO (control) or 150 μM repaglinide. Upper panel: sequential images of sperm flagellar waveforms at 5 ms-intervals. Lower panel: changes of flagellar curvature are plotted against the distance from the base of flagellum. 40 (A) or 20 (B) waveforms produced at 200 ms (A) or 100 ms (B) are overwritten.

**
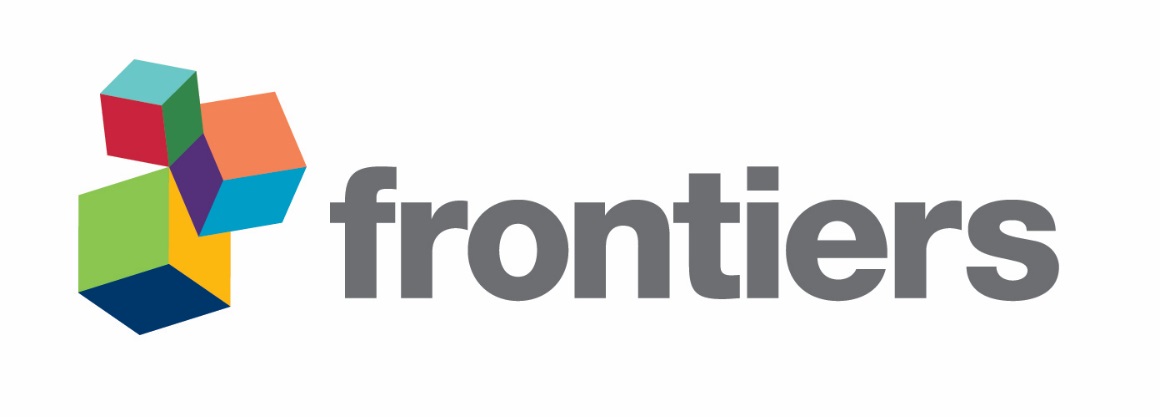
**
